# A Four-Year-Old Female With a Rare P53 Gene Mutation Diagnosed With Li-Fraumeni Syndrome and Concomitant Metastatic Rhabdomyosarcoma: A Case Report

**DOI:** 10.7759/cureus.27009

**Published:** 2022-07-19

**Authors:** Umberto M Donato, Sebastian Donato, Andrew Galligan

**Affiliations:** 1 Health Outcomes and Behavior/Radiology, Moffitt Cancer Center, Tampa, USA; 2 Pediatric Oncology, Tampa General Hospital, Tampa, USA; 3 Pediatric Hematology/Oncology, University of South Florida (USF) Health, Tampa, USA; 4 College of Food, Agricultural, and Environmental Sciences, The Ohio State University, Columbus, USA; 5 Pediatric Oncology, Moffitt Cancer Center, Tampa, USA; 6 Pediatric Hematology/Oncology, University of South Florida (USF) Morsani College of Medicine, Tampa, USA

**Keywords:** genetic syndromes, rhabdomyosarcoma (rms), radiation and clinical oncology, tumor suppressor protein p53, li-fraumeni syndrome

## Abstract

Li-Fraumeni syndrome (LFS) is an autosomal dominant disorder that often results from mutations that impair the functions of the tumor suppressor gene p53. LFS is categorized as a hereditary cancer predisposition syndrome in which patients frequently suffer from an elevated degree of onset and incidence of neoplastic malignancies. Among the different pathogenic variants of LFS, TP53 is one of the most frequently encountered ones. A four-year-old female is reported in this vignette, with a rare c.375+1G>T pathogenic variant in the TP53 gene consistent with an LFS diagnosis. To our knowledge, this is the first reported “germline” example of this variant in the literature. Initially, the patient presented to the emergency department due to concerns of progressive swelling and firmness of a mass in the patient’s right abdomen. Further imaging and analysis revealed a rhabdomyosarcoma of the pelvis secondary to LFS. In addition to this, the patient's brother and mother both were positive for the same LFS mutation allowing us to make a definitive LFS diagnosis. Our patient then underwent neoadjuvant chemotherapy, radiotherapy, and eventually a resection of the main neoplastic lesion. Among pediatric LFS patients, the risk of suffering secondary and/or multiple cancers is pathologically elevated. That said, it is crucial to perform genetic analysis tests for pediatric oncology patients, especially those patients with hereditary predisposition to cancers. Considering the poor prognosis of most TP53 mutations, it is of utmost importance to implement prompt and systematic care for patients diagnosed with LFS.

## Introduction

Li-Fraumeni syndrome (LFS) is an uncommon genetic cancer syndrome characterized by autosomal dominant mutations of the TP53 tumor suppressor gene. This pathology was first cited in 1969 by Li and Fraumeni after studying a subset of families that appeared to have a high frequency of soft tissue neoplastic disease [1]. Be that as it may, it was not until two decades after that the etiology of the diseases was understood. That said, LFS is frequently seen in families in which several individuals can be affected and are at an increased risk of developing several primary tumors. The probability of developing primary cancer in LFS increases steadily with age. For example, by the age of 30 years, this probability is 49% and 21% in females and males, respectively [2]. Moreover, the cumulative probability of developing a secondary neoplasm is close to 15% in both sexes [2]. In a University of Texas study in 2003 of 56 p53 germline LFS carriers, the most common cancerous lesions were soft tissue sarcomas and breast cancers [3]. Soft tissue sarcomas, specifically rhabdomyosarcomas (RMSs) are the most common LFS cancers in the pediatric population and account for approximately 17-27% of the totality of cancers in LFS patients [4]. RMSs are in fact one of the few neoplasms where the cumulative risk for men is higher than that of women [5]. Moreover, RMSs appear to be more prevalent in LFS carriers aged five years or less [6]. Altogether, we present a case of a metastatic RMS secondary to a rare germline LFS mutation.

## Case presentation

**Figure 1:**
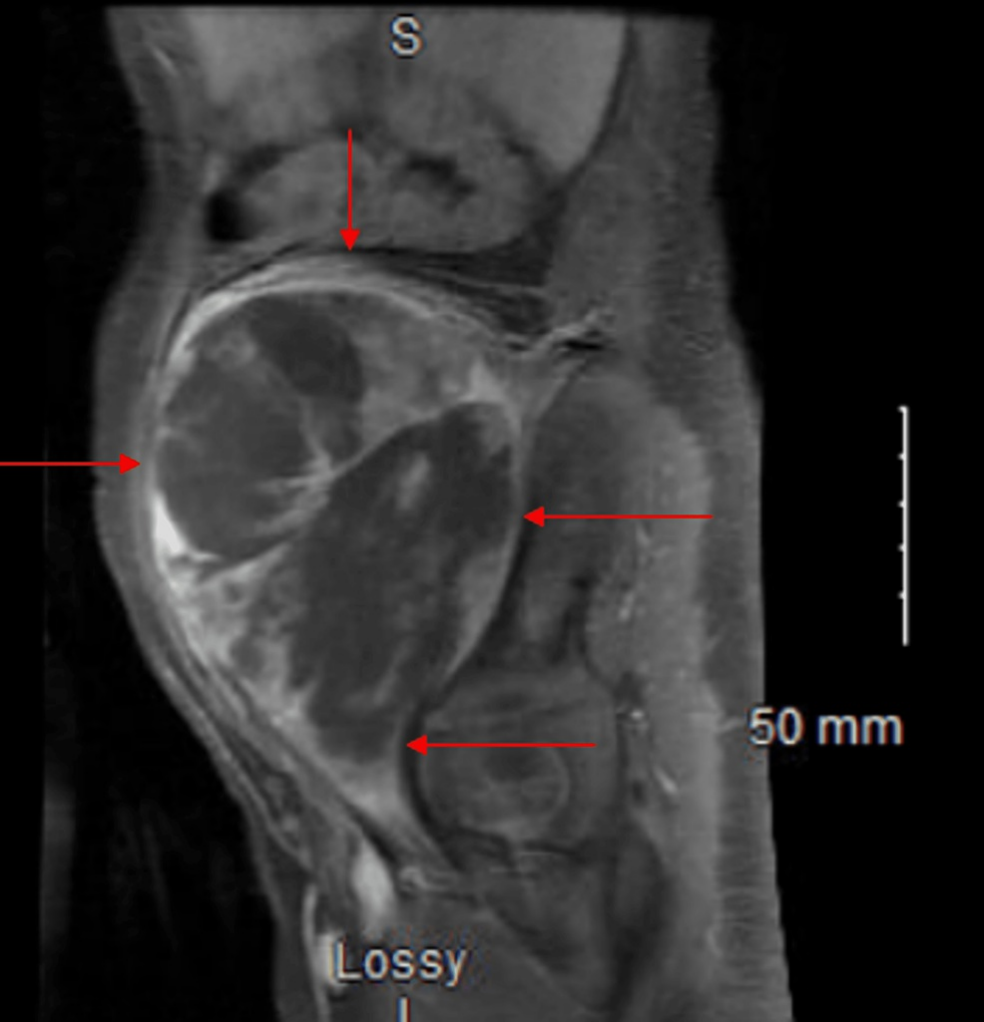
Sagittal T1-weighted MRI without contrast Rhabdomyosarcoma (RMS) is delineated by arrows in imaging.

**Figure 2:**
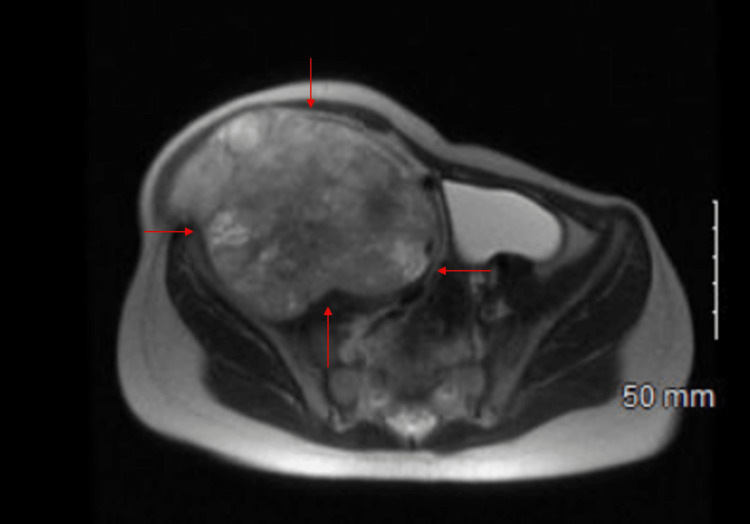
Axial T2-weighted MRI without contrast Rhabdomyosarcoma (RMS) is delineated by the arrows.

A four-year-old female presented to her primary care provider due to the presence of a right lower quadrant abdominal mass that appeared to be “growing” according to the patient’s mother. The mother stated that the patient was not in any pain and denied nausea, vomiting, diarrhea, constipation, and fever. Complete blood count/comprehensive metabolic panel (CBC/CMP) laboratory results were unremarkable. An ultrasound was then performed which revealed a solid mass that appeared to be either ovarian or lymphoid in nature. After the ultrasound evaluation, the patient was sent to the ED for further analysis. The mother mentioned there was a significant family history of Li-Fraumeni syndrome in which the patient’s siblings and herself both were diagnosed with LFS. In addition to this, the mother and the patient’s aunt both had a history of breast cancer in the setting of an LFS mutation. She was unable to recall the specific LFS mutation she carried and was referred to undergo genetic testing along with her daughter (patient) and sister. Genetic testing showed the aforementioned family members shared a rare c.375+1G>T pathogenic variant in the TP53 gene consistent with an LFS diagnosis. Once at the ED, the patient underwent MRI and CT scans along with an image-guided needle biopsy. The CT/MRI analysis (without contrast) was notable for an 8.8 x 11.0 x 15.1 cm heterogeneous mass within the right iliopsoas muscle (Figures 1, 2).

The mass extended from the level of the right inferior renal pole to the right femoral head. There was also a persistent mass effect on intra-abdominal structures including the bowel and urinary bladder, which were largely distended. The right iliac vessels and inferior vena cava (IVC) were effaced by the mass. There were mildly enlarged right superficial inguinal lymph nodes measuring up to 1.5 x 0.9 cm. These were likely related to more proximal lymphatic obstruction. No osseous invasion or suspicious osseous lesions were seen as well. Overall these imaging studies were suspicious for a right iliopsoas rhabdomyosarcoma with intra-abdominal extension. This differential was confirmed when the biopsy results of the neoplasm arrived. The biopsy presented a malignant tumor with cells that appeared grossly to be prominently spindled, anaplastic, some with intranuclear vacuoles. These cells also had a highly eosinophilic cytoplasm and prominent rhabdoid morphology. Immunohistochemical analysis was performed with appropriately reactive controls.

The aforementioned malignant cells were positive for desmin, vimentin, myogenin (focal), myoD1, pancytokeratin and negative for MDM2 and P53. The morphology, size, and immunohistochemical data proved to be consistent with a rhabdomyosarcoma, likely embryonal stage IIIB, group 3; the degree of cellularity was however reduced in comparison to reference embryonal RMSs. Moreover, due to the size and location of the tumor, surgery was not immediately feasible. The patient was started on a chemotherapy protocol COG ARST0431 with possible surgery and definite radiation in the future. Within this regimen, week four and week seven were switched due to planned radiation therapy. This was done in order to prevent the toxicity effects of doxorubicin/cyclophosphamide associated with concurrent radiation. A month into the chemotherapy regimen, the results were suboptimal being that the tumor had not decreased in size. The patient was then switched to an ARST 0121 protocol.

**Figure 3:**
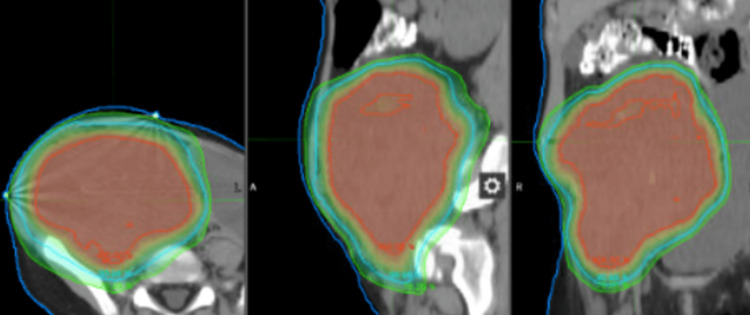
Intensity-modulated radiotherapy target volumes Planned treatment volumes - 54Gy (green), 60Gy (blue), 64 Gy (red).

As far as the radiation treatment goes, the patient received 30 fractions of intensity-modulated radiotherapy (IMRT) in order to shrink the tumor in preparation for potential surgery. IMRT treatment was used despite the fact that it augmented the integral dose to the patient. Since getting a response for the tumor outweighed the increase in long-term risk associated with this approach at this time, the radiation oncologist opted for IMRT. The planning target volume for 54 Gy of radiation is worth noting since it was important to exclude most of the bladder and pelvic bones as possible (Figure 3).

Once IMRT was done, the patient continued the ARST 0121 protocol and underwent resection of the RMS, and continued the chemotherapy regimen post-op. Tumor biopsy results noted an absent FOXO1 rearrangement and negative tumor margins. FOX01 rearrangements are typically seen in alveolar RMS. Nonetheless, immunohistochemistry was consistent with the initial CT-guided biopsy and with the history of treated rhabdomyosarcoma. Be that as it may, there was marked nuclear atypia. This element along with the absence of p53 staining, 10% tumor necrosis, and prior neoadjuvant therapy favored this to be treatment-related atypia. Additional cephalad margins of the resection were also negative for the tumor. Overall, the resection procedure went as expected (no complications) and the patient was continued on her chemotherapy regimen post-op.

## Discussion

A variety of genetic conditions can predispose certain individuals to the development of pelvic rhabdomyosarcomas (RMSs). The vast majority of the literature pertinent to RMSs, however, does not reference the location or etiology of these neoplasms. That said, the data on the specific associations of retroperitoneal-pelvic neoplasms with particular genetic syndromes is very limited. Approximately 25% of individuals diagnosed with LFS will develop a sarcoma during their lifetime, with RMS being the most common sarcoma in LFS [7]. With regards to our patient, she was diagnosed with a malignant embryonal RMS secondary to an LFS germline mutation. Non-embryonal neoplasms associated with LFS such as the one in our patient, typically have a worse prognosis than their embryonal counterparts [7]. Knowing this, providing an accurate diagnosis for our patient was of the utmost importance. Our patient met the diagnostic requirements of LFS otherwise known as the Chompret criteria - a tumor belonging to the LFS tumor spectrum (sarcoma) before 46 years of age, at least one first- or second-degree relative with an LFS tumor before 56 years of age, and positive TP53 genetic testing [8]. Despite the positive Chompret criteria, genetic testing was still required in order to have a better understanding of our individual patient’s condition. The genetic testing results were quite unique. It has been noted that the p53 gene on chromosome 17, codon 175 is a mutational hot spot for LFS [9]. Despite this and the fact that a variety of different mutations of the p53 tumor suppressor gene have been cited in the literature, our patient’s p53 mutation (c.375+1G>T pathogenic variant) did not take place in this “hot spot.”

The aforementioned variant has been reported in plasma-derived circulating tumor DNA (ctDNA) from an individual with lung cancer and multiple metastases, but to the best of our knowledge, it has not been reported as a germline variant [10]. Regarding the diagnosis of the embryonal RMS, immunohistochemical analysis was vital. The tumor’s desmin, vimentin, MYOD1, and myogenin immunoreactivity was pathognomonic to an embryonal RMS [11]. Further histological analysis was also notable for reduced tumor cellularity in comparison to reference embryological RMSs and an abundance of long fascicles of spindled rhabdomyoblasts. These histopathologic characteristics were consistent with an example of “spindle cell RMS” [12]. This tumor type is very rare and may be responsible for its suboptimal response to chemotherapy seen in different cases [12].

Altogether, considering the elevated incidence of p53 mutations in children (less than five years of age) with rhabdomyosarcomas, it is reasonable to assume that Li-Fraumeni syndrome is present and not identified in certain cases of pelvic RMS [4]. With respect to our case, attaining a formal oncologic family history served to promptly provide us with an LFS differential. The significant family history of cancer in this patient, complemented with immunohistochemical analysis and genetic testing, allowed us to rapidly and definitively provide an LFS diagnosis and thus provide adequate systematic care to our patient.

## Conclusions

Prompt identification of LFS via genetic testing and taking a thorough family history can improve treatment options by allowing physicians to adequately weigh the benefits of utilizing radiation and/or aggressive surgical resection in cases of RMSs secondary to an LFS diagnosis. Furthermore, our patient’s extremely uncommon P53 mutation can serve to document and highlight the progression, prognosis, and standard of care for similar LFS patients in the future.
